# “Ultrasound-Assisted High-Fluidics Phacoaspiration”: An Efficient and Safe Technique for Nucleus Quadrant Workup Through 2.2 mm Mini- and 1.6 mm Micro-Incisions Following Manual or Femtosecond Laser Sectorial Lens Fragmentation

**DOI:** 10.3390/jcm14165887

**Published:** 2025-08-20

**Authors:** Rupert Michael Menapace, Sabine Schriefl, Silvio Di Nardo

**Affiliations:** 1 Department of Ophthalmology, Medical University of Vienna, A-1090 Vienna, Austria; 2 Institute of Medical Engineering, Lucerne University of Applied Sciences and Art, CH-6048 Horw, Switzerland; silvio.dinardo@hslu.ch

**Keywords:** coaxial longitudinal phacoemulsification, high fluidics, slim-shaft-strong-bevel tip design, infusion-assisted (Hybrid) micro-phacoemulsification

## Abstract

**Purpose**: To evaluate the efficiency and safety of high-fluidics ultrasound emulsification using slim-shaft-strong-bevel (SSSB) tip technology for nucleus workup through mini- and micro-incisions. **Materials and Methods**: 77 patients underwent immediate sequential bilateral cataract surgery using high-flow-high-vacuum easyTip^®^2.2 (“eT2.2”) or infusion-assisted (“Hybrid”) easyTip^®^COMICS (“eT1.6 iaCOMICS”) coaxial phacoemulsification. Surgical time (ST), Effective Phacoemulsification Time (EPT), and fluid consumption (FC), central corneal thickness (CCT), and endothelial cell count (ECC) were recorded. **Results**: 50 patients completed the 6 months follow-up. Preoperatively, groups did not differ in nuclear hardness, CCT, or ECC. The median ST for Conquer with eT2.2 phaco was 49 s, with eT1.6 iaCOMICS phaco 57 s (*p* = 0.021). The median total EPT was 8.3 and 8.0 (*p* = 0.882), and the median EPT for Conquer was 4.0 and 4.1, respectively (*p* = 0.812). The median FC for Conquer was 21 mL with the eT2.2 and 22 mL with the eT1.6 iaCOMICS phaco (*p* = 0.29), and the overall FC was 29 mL and 33 mL, respectively (*p* = 0.105). Mean CCT was 561 ± 42 µm and 563 ± 45 µm on day 1 (*p* = 0.835), 539 ± 33 µm and 542 ± 34 µm at 1 week (*p* = 0.714), and 536 ± 31 µm and 541 ± 33 µm at 6 months (*p* = 0.55), respectively. Mean ECL at 6 months was 2.80 ± 7.28% and 3.41 ± 8.25% (*p* = 0.725). **Conclusions**: When compared to previously published results obtained with a standard non-waisted phaco needle and lower fluidics and with the waisted easyTip^®^2.2 run with intermediate fluidics, ultrasound-assisted high-fluidics phacoaspiration with the easyTip^®^2.2 through a 2.2 mm incision and the easyTip^®^ COMICS through a 1.6 mm incision with infusion-assistance significantly improved efficiency of nucleus workup by reducing ultrasound energy and infusion fluid consumption, with minimal transient corneal stroma swelling and low endothelial cell loss.

## 1. Introduction

Femtosecond laser (FSL) pre-fragmentation may help reduce ultrasound (US) energy consumption for emulsification of the cataractous lens. However, the aspiration of the fragments still requires US energy, particularly with those FSLs that work with quadrant or other sector patterns. Phacoemulsification, or “phaco”, has continuously evolved over time regarding both machine and needle technology. Transversal oscillation introduced by Alcon (Fort Worth, TX, USA: OZil^®^ torsional phaco), and conventional longitudinal oscillation with slim-shaft-strong-bevel (SSSB) needle technology favored by Oertli Instruments (Berneck, Switzerland) in conjunction with improved pump technologies have been the main advancements.

Phaco efficiency is defined by the time, US energy, and infusion fluid required for emulsifying and aspirating a cataractous lens with a nucleus of defined hardness. Phaco safety is defined as chamber stability particularly in the event of an instant occlusion break and consecutive surge, and the effect of US on ocular tissue, specifically on the corneal endothelium. SSSB phaco needle technology aims at fully exploiting the potential of longitudinal needle oscillation. In order to achieve this, high flow (45–50 mL/min) and vacuum settings (500–600 mmHg) are used. High vacuum levels ensure strong power coupling and thus efficient energy transfer during phaco, thereby reducing the total amount of US energy dispersed. High flow readily attracts nuclear chunks to the needle orifice and allows the preset vacuum level to be more quickly attained when occluded, or re-attained in the case of intermittent partial occlusion break, thus ensuring a quick and firm grasp, or re-grasp, of the nuclear material.

Holdability, however, is not solely a function of the actual vacuum, but also of the area of the needle orifice, and energy transfer is not only a function of the longitudinal stroke length, but also of the area of the frontal projection of the tip. To profit from both, the bevel of the needle tip was increased, and the diameter of the shaft and bore reduced, with a stepped outer and inner transition (waisted “slim-shaft-strong-bevel” or SSSB phaco needle design).

When the occlusion breaks, the released vacuum induces surge. This is caused by the inherent compliance load of plastic aspiration tubing. In spite of significant improvements by implementing stiffer materials and thicker walls, modern aspiration tubing still retains some elasticity and, thus, compliance. Though only minimally contracting under high vacuum, the stored elastic energy is abruptly discharged when occlusion breaks, causing an aspiration peak (surge) which results in chamber flattening or even collapse if it exceeds the infusion influx capacity. Surge may be counterbalanced by the pressure sensor-controlled reverse action of the pump but can also be suppressed by increasing the flow resistance in the aspiration line by reducing the bore diameter of the shaft of the phaco needle (Hagen-Poiseuille’s law). While the reverse action is inherently delayed due to pump reaction time, the flow resistance of a slim shaft bore sets in immediately.

**Figure 1:**
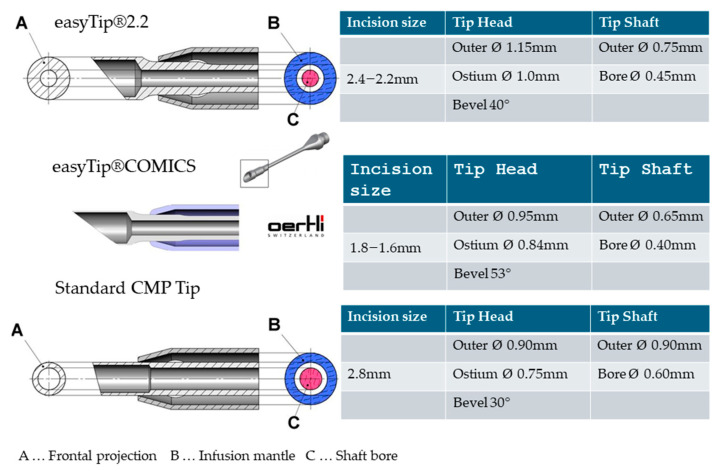
The easyTip^®^2.2 and the easyTip^®^COMICS phaco needles have a swollen head and a stepped transition into a slim shaft (“waisted” tip design) while head and shaft of the traditional-design CMP^®^ (Cool Microincision Phaco) 2.8 mm needle (Oertli Instruments, Berneck, Switzerland) for standard low-flow phacoemulsification feature a continuous outer diameter (“non-waisted” tip design). The recommended incision size depends on the incision architecture, clear corneal incisions requiring a somewhat larger incision than posterior limbal incisions. The slim shaft counteracts surge by the increased aspiration flow resistance (small shaft bore) while decreasing that of the infusion flow (widened infusion mantle). The strong bevel increases the area of the orifice (holdability), the stepped transition of the wide tip into a small shaft bore that of the frontal projection (energy output).

In scientific cooperation with the principal author, the Oertli Instruments Team for Research and Development has worked on a SSSB needle design (now marketed as easyTip^®^) that should combine all the aforementioned characteristics (Figure 1). The outer contour of the thin-walled coaxial silicone sleeve runs almost flush with that of the swollen needle head and seals off with the shaft-to-head transition. This prevents forward directed infusion flow that interferes with the aspiration flow and the regular flow path between sleeve outlets and tip orifice. The large diameter and strong bevel of the needle head add up to a 2- and 3-fold increase in orifice area and thus holdability compared to the previous non-waisted phaco needles from the company. The stepped lumen transition from needle head to shaft increases the frontal projection area and, thus, energy emission by a factor of five for the easyTip^®^2.2 and six for the easyTip^®^COMICS tip (Oertli Instruments, Berneck, Switzerland), respectively. The small shaft bore suppresses surge upon loss of occlusion, as does the correspondingly increased infusion supply through the widened infusion mantle along the slim shaft. This allows for maximum vacuum settings in order to provide maximum holdability and power-coupling while suppressing surge when occlusion breaks. The high infusion influx capacity provided by the low flow resistance of the broad infusion mantle along the slim needle shaft provides high flow rates, which improves followability and accelerates vacuum rise. Altogether this should result in an increase in efficiency and safety of phacoemulsification as mirrored by a decrease in the time and energy required, a rock-solid chamber, and low corneal swelling and endothelial cell loss rates.

This paper reports on the efficiency and safety of Oertli easyTip^®^ SSSB tips for mini- and micro-phacoemulsification through 2.2 and 1.6 mm incisions using high fluidics settings, the latter combined with an infusion spatula inserted through a sideport to augment infusion influx.

## 2. Materials and Methods

A total of 77 patients were included in this prospective, randomized intraindividual comparison. One eye was assigned to have high fluidics phaco with an easyTip^®^2.2 mini-needle through a 2.2 mm incision (eT2.2 group) and the other eye with an easyTip^®^COMICS micro-needle with sideport infusion assistance through a 1.6 mm incision (eT1.6 iaCOMICS group).

The study was performed at the Department of Ophthalmology at the Vienna General Hospital (Medical University of Vienna, Austria). The patients were recruited in a continuous cohort. Inclusion criteria were bilateral age-related cataract, eligibility and willingness to undergo immediate sequential cataract surgery (ISBCS), and good overall physical constitution. Exclusion criteria were a history of intraocular surgery, glaucoma, inflammatory eye disease, any corneal disease, and severe retinal pathology that would make a postoperative visual acuity of 20/40 (decimal equivalent = 0.5) or better unlikely. The study was approved by the local ethics committee of the Medical University of Vienna, Austria. All the research and measurements followed the tenets of the Helsinki agreement and informed consent was obtained from all subjects in this study.

Preoperative examinations took place 1 week before cataract surgery and included cataract grading using the Lens Opacities Classification (LOCS) III, endothelial cell count (ECC), and central corneal thickness (CCT) measurement.

Surgeries were performed by one surgeon (R.M.). One eye was randomly assigned to group eT2.2 and the other eye to group eT1.6 iaCOMICS.

An Oertli OS3 peristaltic pump phaco machine was used. Ringer solution and methylcellulose (Hypromellose 2.0%) were used as infusion fluid and ophthalmic viscoelastic device (OVD) during phacoemulsification.

Machine settings for quadrant conquer were as follows: Linear phaco power maximum 70%, vacuum limit 500 and 600 mmHg at a bottle eight 100 cm, and flow at 45 and 50 mL/min for the eT2.2 and eT1.6 iaCOMICS groups, respectively [see Table 1]. Surgical technique was as follows [1,2]: With the surgeon seated on the temporal side, a square, internally flared posterolimbal incision was created with a thin bevel-up metal blade, 1% preservative-free lidocaine was injected intracamerally, and the aqueous exchanged for methylcellulose. Two sideports were added supero- and infero-temporally.

**Figure 2:**
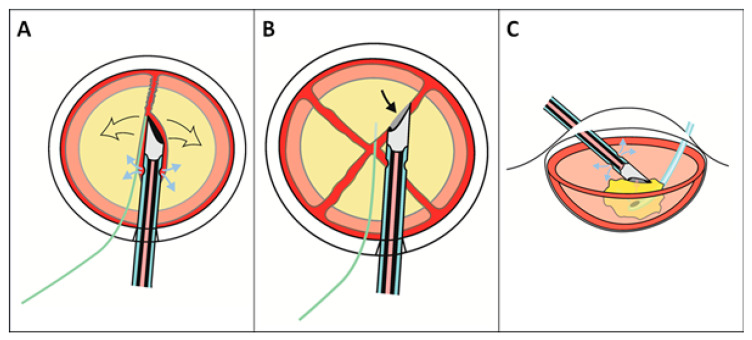
Steps of the modified Divide-and-Conquer (“Crack-and-Conquer”) technique. “Direct cracking” used with SSSB tip: The grooving step is omitted. Instead, with bevel turned sideward, the tip deeply penetrates the nucleus core for subsequent cracking of cataractous lens with a special spatula (Bausch&Lomb REF 55485). (**A**). Cracking manoeuvre (**B**). The paddle-shaped spatula maneuvers quadrant towards sideward-oriented phaco tip orifice to promote ready occlusion. (**C**). During high-fluidics phacoaspiration, the spatula protects endothelium and posterior capsule from nucleus material or tip contact.

Following capsulorhexis, thorough hydrodissection was performed and the lens rotated for 360° to spin the periphery completely free from all cortico-capsular adhesions. The lens was divided into four quadrants using the following technique (surgeon right-handed):

The sleeve outlets were aligned with the tip bevel. The bevel was oriented sideward to the right and the tip directed downward to the very center of the nucleus while using only as much US energy as necessary to penetrate into the nucleus core. With occlusion achieved, the tip of a slim paddle-shaped spatula (Menapace Nucleus Divider, REF 55485, Bausch&Lomb, Rochester, NY, USA) was now advanced into the trough alongside the left flank of the phaco needle while the latter stabilizes the nucleus by vacuum. When arrived at the end of the phaco needle, the spatula was moved to the left, thereby creating a crack in the central and distal portion of the nucleus (Figure 2A). The cleft thus created was then extended with the spatula inferiorly to the posterior lens pole and proximally until the whole lens was completely separated into two halves. The lens was then rotated counterclockwise for a quarter-turn and the nasally positioned first lens half engaged and split into two quadrants as described. After another half-turn the second half was similarly divided into two quadrants. Again, care was taken to fully separate the quadrants thus created. The dividing procedure described was similar to that used by the author after FSL sectorial pre-fragmentation.

For quadrant aspiration, fluidics were adjusted to the particular tip used:

In group eT2.2, flow and vacuum were increased to 45 mL/min and 500 mmHg. The paddle-shaped spatula was used to tilt up and maneuver the quadrants towards the still sideward-oriented tip orifice in order to promote ready occlusion (Figure 2B). Only now was the pump activated to engage the lens material using minimal phaco energy to initiate circumferential sealing of the needle orifice. When full vacuum was signaled acoustically, only the minimal ultrasound energy necessary to start and maintain aspiration of the engaged nuclear chunk was used, aiming at what is best termed as minimal-energy “ultrasound-assisted phacoaspiration” instead of standard high-energy US phacoemulsification. During high-fluidics phacoaspiration, the spatula was held above or below the nuclear chunk to prevent endothelial contact by a tilting-up or tumbling nuclear chunk or capsular damage by phaco tip contact, respectively (Figure 2C).

In group eT1.6 iaCOMICS, an additional infusion handpiece or spatula was introduced through the left sideport to compensate for the reduced influx capacity of the easyTip^®^COMICS needle which is only half the amount of the eT2.2 needle (approx. 40 mL/min instead of 80 mL/min at a bottle height of 100 cm) because of the smaller size and thus smaller cross-section of the infusion mantle. The influx capacity of the 20-gauge Oertli biaxial I&A infusion handpiece is 50 mL/min, complementing the coaxial influx of the easyTip^®^COMICS needle to a total of 90 mL/min. This allows the pump to be set at its maximum of 50 mL/min flow and 600 mmHg vacuum limit while still guaranteeing a rock-solid chamber during aspiration and after instant occlusion break. The rounded tip of the irrigation handpiece used allows for gentle manipulation and guidance of the nuclear quadrants and chunks, and for protection of both the endothelium and posterior capsule.

Maximum linear phaco power, vacuum limits, and flow rates used with groups eT2.2 and eT1.6 iaCOMICS are summarized in Table 1.

With phacoaspiration of the nucleus completed, the residual cortical material was removed using coaxial aspiration through the phaco incision in Group eT2.2, and biaxial aspiration through sideport openings in Group eT1.6 iaCOMICS, and a foldable intraocular lens (IOL) was injected with the injector tip inserted in the 2.2 mm incision or docked to the 1.6 mm incision. Following OVD removal, the sideports were hydrated, 0.1 mL cefuroxime 1% was injected, and the globe tonified.

In this series, the main incision was spontaneously self-sealing in all cases without the need of additional hydration. No case of thermal tissue burn or shrinkage of, or around, the main incision was observed. As a postoperative regimen, patients applied prednisolone acetate (Ultracortenol) and ketorolac-trometamol drops (Acular) 3 times daily for 3 weeks.

Surgical Time (ST) equivalent to “needle in the eye”-time, Effective Phaco Time (EPT) representing the integral of phaco energy over time, and fluid consumption (FC) were recorded separately for the divide (crack) and conquer procedures, respectively.

Patients were examined 1 day, 1 week, and 6 months post operation. Examinations included best-corrected distance visual acuity as well as CCT and ECC measurements.

Data are presented as means ± standard deviation (SD) if normally distributed or as medians [Minimum; Maximum] if not. Numeric data were analyzed using *t*-test if normally distributed or Mann-Whitney Test if not. A *p*-value of 0.05 or less was considered significant.

## 3. Results

A total of 77 patients aged 75 ± 7 years were recruited for the study (60% females). A total of 50 patients completed the 6 month follow-up. Mean age of these patients was 74 ± 7 years.

Preoperatively, nuclear hardness graded according to the Lens Opacification System LOCS III [3] was comparable in both groups (median 2 [2; 4] vs. 3 [2; 4], *p* = 0.835 Mann-Whitney).

**Figure 3:**
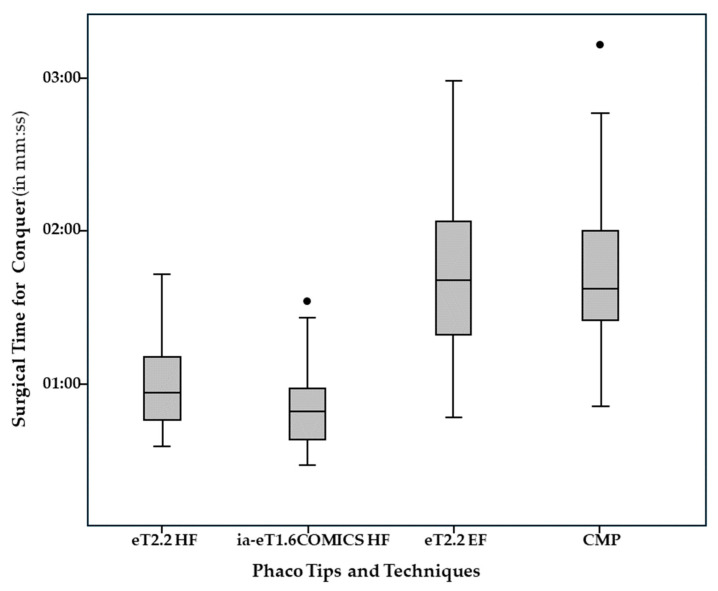
Surgical (Needle-in-the-Eye) Time as a measure of overall efficiency of the surgical procedure for high-fluidics easyTip^®^2.2 (eT2.2 HF) and high-fluidics infusion-assisted (Hybrid) easyTip^®^COMICS (eT1.6 iaCOMICS HF), compared to standard enhanced-fluidics waisted SSSB-tip (eT2.2 EF) and to low-fluidics conventional-tip (CMP^®^) 2.8 mm coaxial phaco. DOTs represent mild outliers (Data volume lies more than 1.5 times the interquartile range above the third or below the first quartile).

**Figure 4:**
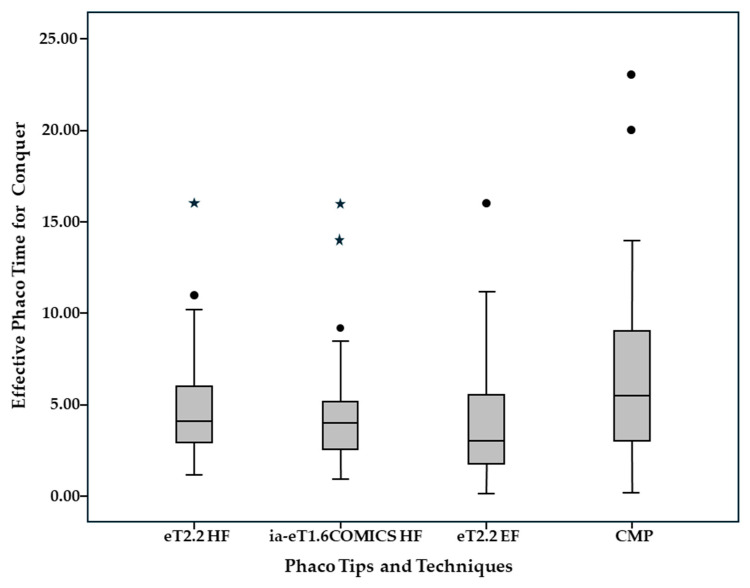
Effective Phaco Time for Conquer as a measure of phaco energy required for nucleus work-up for high-fluidics easyTip^®^2.2 (eT2.2 HF) and high-fluidics infusion-assisted (Hybrid) easyTip^®^ COMICS (eT1.6 iaCOMICS HF), compared to standard enhanced-fluidics waisted SSSB-tip (ET2.2 EF) and to low-fluidics conventional-tip (CMP^®^) 2.8 mm coaxial phaco. DOTs represent mild outliers, ASTERISKs represent extreme outliers (Data volume lies more than 3 times the interquartile range above the third or below the first quartile).

**Figure 5:**
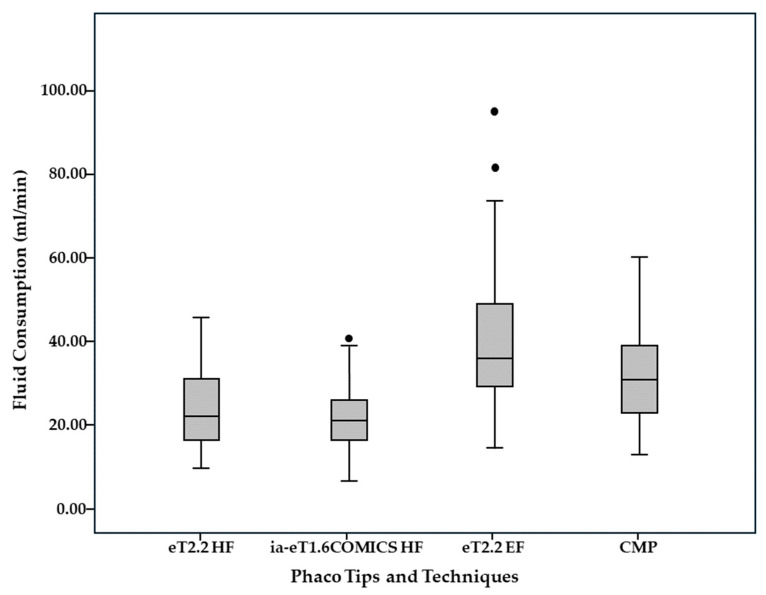
Fluid Consumption for Conquer as a measure of infusion fluid throughput during nucleus work-up for high-fluidics easyTip^®^2.2 (eT2.2 HF) and high-fluidics infusion-assisted (Hybrid) easyTip^®^ COMICS (eT1.6 iaCOMICS HF), compared to standard enhanced-fluidics waisted SSSB-tip (ET2.2 EF) and to low-fluidics conventional-tip (CMP^®^) 2.8 mm coaxial phaco. DOTs represent mild outliers.

Time, energy, and fluid consumption results are listed in Table 2. Median overall ST for Divide and Conquer was significantly lower in the eT2.2 group (1 min 29 s [57 s; 2 min 12 s]) than in the eT1.6 iaCOMICS group (1 min 40 s [1 min 9 s; 2 min 30 s]) (*p* = 0.011). Median ST for Divide alone was not statistically significantly different, while median ST for Conquer was again significantly lower in the eT2.2 than in the eT1.6 iaCOMICS group—49 s [28 s; 1 min 32 s] compared to 57 s [35 s; 1 min 43 s] (*p* = 0.021) (Figure 3). Median total EPT as well as median EPT for Divide and median EPT for Conquer (Figure 4) were comparable for both groups. As with EPT, total fluid consumption as well as FC for Divide and FC for Conquer (Figure 5) were also comparable in both groups. Best-corrected median Log MAR visual acuity at 6 months was 0 in both groups ([0; 0.20], and [0; 0.30], respectively, *p* = 0.255). 

Preoperative values and postoperative development of corneal parameters are listed in Table 3.

Preoperative values were comparable in both groups. The almost identical mean preoperative CCT in both groups rose statistically significantly after surgery (day 1) but regained baseline values at the 1 week and 6 months follow-up, with no statistically significant difference found between groups at any point in time.

**Figure 6:**
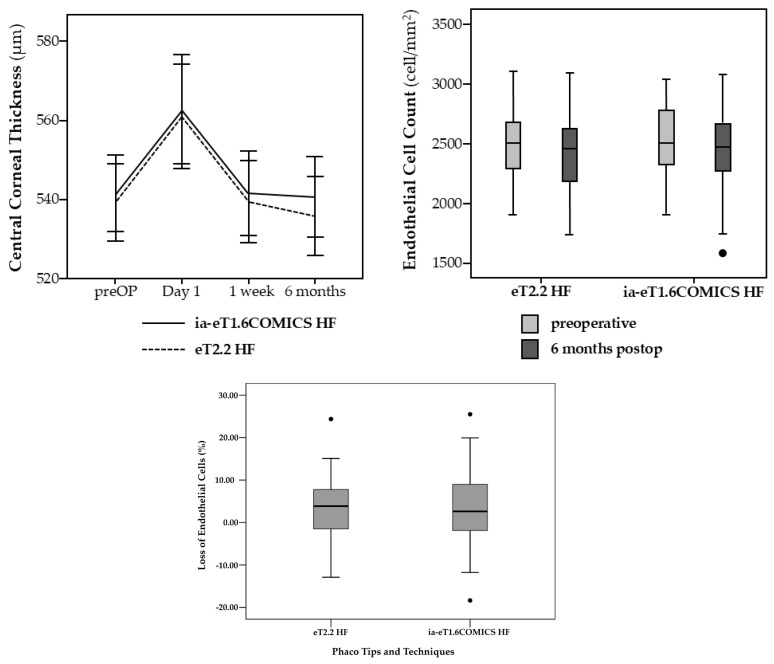
With both eT2.2 HF and eT1.6 iaCOMICS, central corneal thickness regained baseline values at one week, with no statistically significant difference between groups at any point in time. Endothelial cell loss was 2.8 and 3.4% in 6 months, respectively. DOTs represent mild outliers.

No statistically significant difference was found in ECC at 6 months postoperatively (Figure 6). Mean endothelial cell loss (ECL) at 6 months was 2.80 ± 7.28% in the eT2.2 group, and 3.41 ± 8.25% in the eT1.6 iaCOMICS group (*p* = 0.725) (Figure 6).

## 4. Discussion

The aim of innovations in medical technologies is to improve the efficiency and safety of procedures. For phacoemulsification this translates into a reduction of surgical (needle in the eye) time, phaco energy consumption or EPT, and fluid throughput while maintaining chamber stability to minimize the risk of capsular and endothelial damage.

The slim-shaft-strong-bevel, or SSSB phaco needles by Oertli were designed to be used with high fluidics and maximize holdability and energy transfer while efficiently suppressing surge.

In a previous intraindividual comparison study we compared the easyTip^®^2.2 operated with standard “enhanced” fluidics (eT2.2 EF group with 35 mL/min, 500 mmHg) to a conventional non-waisted CMP^®^ (Cool Micro Phaco) 2.8 mm tip run with low fluidics (CMP group with 20 mL/min, 400 mmHg [4], Table 4): Nuclear hardness according to LOCS III was similar in both groups. Median EPT for Conquer was significantly reduced by 50% (5.9 in CMP group to 3.0 in eT2.2 EF, *p* = 0.001, see Figure 4) while median fluid consumption only increased by 14% (31.07 mL to 35.97 mL, *p* = 0.016, see Figure 5) in spite of an increase in pump speed by 75% (35 vs. 20 mL/min, resp.). While the reduction of EPT for conquering mirrored a substantial gain in phacoemulsification efficiency, the moderate increase in fluid consumption at an almost doubled pump speed indicated that the proportion of occluded (“no flow”) versus unoccluded (“free flow”) time markedly increased even though the larger orifice of the easyTip^®^2.2 needle is inherently more difficult to occlude.

The current study investigated the performance of the easyTip^®^2.2 mini-phaco needle and easyTip^®^COMICS micro-phaco needle when run with high fluidics (500 mL/min and 45 mmHg versus 600 mL/min and 50 mmHg with fluid assistance, respectively). Benchmarking efficiency (time, energy, and fluid consumption) and safety results (endothelial cell swelling and loss) against the eT2.2 phaco with intermediate instead of high fluidics elucidates the positive effect of further increasing fluidics on SSSB-needle performance. Median EPT for nucleus conquering with the eT2.2 and eT1.6 iaCOMICS groups using high-fluidics was not statistically significantly different from the eT2.2 EF group run with intermediate fluidics. This is not surprising, since the preset maximum vacuum levels were similar in all three groups. However, compared to the eT2.2 EF coaxial group, median fluid consumption for conquering decreased by 42% and 39% from 36 mL to 21 mL in the eT2.2 coaxial and to 22 mL in the eT1.6 iaCOMICS infusion-assisted high-fluidics groups. The reduction of total fluid consumption from 46 mL to 29 mL and 33 mL in the eT2.2 and eT1.6 iaCOMICS group in spite of an increase in pump speed by 29 and 42% (from 35 mL/min to 45 mL/min with eT2.2 and to 50 mL/min with eT1.6 iaCOMICS phaco) compared to eT2.2 EF phaco with enhanced fluidics again proves that the cumulative time of unoccluded pump action (“free-flow”) decreases while occlusion time increases relative to the overall time required for the conquering maneuver when pump speed and flow rate increase.

High flow rates also accelerate vacuum build-up to high preset levels which quickly establishes maximum holdability. This ensures “quick grasp and firm hold” of nuclear pieces which then “melt down” on the tip head as they are “phaco-aspirated” with the minimum linear US-power needed. Optimal power-coupling combined with the high energy output of the SSSB-tip allows for efficient phacoemulsification of even very hard nuclei at very low US power levels.

Effective Phaco Time, or EPT as the integral of US power over time is a commonly used measure for cumulative energy release. Cavitation or shock waves, which depend on the phaco power, or stroke length of the phaco needle, have been attributed possible sources for tissue damage. With the high holdability and thus power-coupling provided by the high vacuum, only very low US power or stroke lengths are required for emulsification. Thus, high-vacuum SSSB-tip phaco not only decreases overall EPT, but also cavitation. The reduced stroke length also avoids chatter at the tip orifice, ensuring sustained power-coupling, and avoids repulsion of nuclear fragments, thus preventing particles from being pushed away and carried along with the fluid stream. These factors may explain the consistently low corneal swelling and endothelial cell loss observed with high-fluidics SSSB-tip phacoemulsification.

In the above-mentioned study comparing conventional non-waisted CMP^®^ 2.8 mm tip phaco with low and waisted eT2.2 mm tip phaco with intermediate fluidics, endothelial cell loss (ECL) was 4.9 and 6.3%, respectively, with no statistically significant difference between the two groups (*p* = 0.696). These cell losses were in good accordance with the ECL of 7.2 ± 4.6 % and 7.1 ± 4.4 % reported by Reuschel et al. [5] for longitudinal and torsional phacoemulsification. Bozkurt and et al. [6] also found no statistically significant differences in central corneal thickness (CCT) after day 1 and 1 week, and in ECL between these two technologies, as did Kim et al. [7] when looking at CCT and central ECC at 30 days post operation. When comparing high and low flow phaco, Das et al. [8] and Chang et al. [9] also found no differences in CCT, and in ECC or ECL, which was confirmed in a meta-analysis by Kuo et al. [10]. However, a highly significant decrease in phaco energy consumption by 50% was reported by Chang et al. [9].

Compared to 6.3% mean ECL in the eT2.2 coaxial group with standard enhanced fluidics settings, mean ECL in the present high fluidics study was almost halved with only 2.8% and 3.4%. This may be attributed to the shortening of “free flow” phases during which the infusion fluid stream from sleeve outlets to tip orifice carries along nucleus particles that may hit the endothelium while the protective dispersive OVD coating is progressively washed out.

Divide-and-Conquer is still very popular. Storr-Paulson et al. [11] found no difference compared to Stop-and-Chop regarding postoperative ECC, endothelial cell morphology, or CCT. The strong bevel of SSSB-tips requires appropriate adaptation of the Divide-and-Conquer technique. With the central crack technique using the spatula described and the sideward-oriented tip bevel, US radiation directed towards the endothelium is minimized. In rabbit studies, no correlation between fluid consumption and ECL was found [12]. However, ECL significantly increased when simulated lenticular debris were added to the fluid [13], while ECL was significantly decreased when a dispersive OVD used [14]. Orienting tip bevel sideward and sleeve openings horizontally, and shortening free-flow-phases, turbulences and particle-loaded fluid stream are minimized and directed away from the endothelial, providing better endothelial protection by avoiding washout of dispersive OVD and direct endothelial bombardment by particles. The low phaco power levels needed and the insulating broad infusion mantle resulting from the slim shaft of the waisted tip minimizes thermal trauma to the incision and surrounding tissues.

When comparing efficiency and safety parameters of high-fluidics phaco conducted with the easyTip^®^2.2 and with the easyTip^®^ COMICS with fluid assistance, no statistically significant differences were found in the outcomes except the surgical time required for nuclear conquering, which was higher in the eT1.6 iaCOMICS group. The latter is explained by the additional effort required to manipulate the nuclear pieces into adequate position for ready engagement by the 53-bevel eT1.6 iaCOMICS orifice before the pump is fully activated.

In conclusion: High-fluidics coaxial phaco with the easyTip^®^2.2 and easyTip^®^COMICS, the latter with fluid assistance through 2.2 and 1.6 mm incisions have proven highly and equally effective and safe. Compared to the non-waisted CMP^®^ conventional tip with low and the eT2.2 phaco tip with intermediate fluidics, the SSSB-tips in conjunction with high fluidics significantly increased followability, holdability and ultrasound energy transfer, thus minimizing fluid turbulences and cavitation while still ensuring a rock-solid chamber by effectively suppressing surge at the level of the tip. This translates into a significant reduction of surgical (“needle in the eye”) time and ultrasound energy (effective phaco time) consumption overall but specifically for the conquering maneuver with a still low fluid consumption as indicators of efficiency, and low rates of corneal swelling and endothelial cell loss reflecting the safety of the technique. The significant gain in efficiency and reduction of endothelial cell loss compared to intermediate fluidics underlines that high fluidics are key for exploiting the potential of SSSB-needle technology. Also, manual workup of a medium-hard cataract was shown to be equally efficient as with sectorial femtosecond laser pre-fragmentation [15]. The key messages of the study and paper are that the SSSB-tip technology 1. must be operated with high fluidics to fully exploit its potential for increased phacoemulsification efficiency while this does not impact safety, and 2. that, compared to when using standard (enhanced) fluidics, endothelial cell loss is significantly reduced with the particular surgical technique described.

## Figures and Tables

**Table 1:** Machine settings and surgical parameters for high-fluidics easyTip^®^2.2 (eT2.2 HF) and high-fluidics infusion-assisted easyTip^®^ COMICS (eT1.6 iaCOMICS HF) phaco.

Group	“eT2.2 HF”	“eT1.6 iaCOMICS HF”
Phacoemulsification tip	easyTip^®^2.2 Mini-Phaco	easyTip^®^COMICS infusion-assisted (Hybrid) Micro-Phaco
Incision	2.2 mm posterolimbal	1.6 mm posterolimbal
Settings for Crack maneuver	Aspiration flow: 10 mL/min Vacuum limit: 150 mmHg Bottle height: 70 cm above the patient’s eye Power limit/mode: 70%/continuous mode	
Settings for Conquer		
Aspiration flow	45 mL/min	50 mL/min
Vacuum limit	500 mmHg	600 mmHg
Bottle height	100 cm	100 cm
Infusion supply	coaxial only	coaxial + additional infusion via infusion handpiece of biaxial I&A set

**Table 2:** Nuclear hardness, and consumption of time, energy, and fluid for high-fluidics easyTip^®^2.2 (eT2.2 HF) and high-fluidics infusion-assisted easyTip^®^ COMICS (eT1.6 iaCOMICS HF) phaco.

	eT2.2 HF	eT1.6 iaCOMICS HF	*p*-Values
**LOCS III**, Nuclear hardness	2 [2; 4]	3 [2; 4]	0.836
**Surgery time (ST)**			
for Divide and Conquer	1 min 29 s [57 s; 2 min 12 s]	1 min 40 s [1 min 9 s; 2 min 30 s]	**0.011**
for Divide	39 s [25 s; 1 min 16 s]	42 s [27 s; 1 min 29 s]	0.102
for Conquer	49 s [28 s; 1 min 32s]	57 s [35 s; 1 min 43 s]	**0.021**
**Effective Phaco Time (EPT)**			
for Divide and Conquer	8 [3.9; 22.9]	8.3 [2.8; 22]	0.882
for Divide	4.2 [1.8; 12]	3.8 [1.7; 6.5]	0.41
for Conquer	4 [0.9; 16]	4.1 [1.1; 16]	0.812
**Fluid Consumption (FC)**			
for Divide and Conquer	29.4 [14.7; 47.4]	32.7 [16.4; 62.1]	0.105
for Divide	6.5 [3.3; 23]	8.2 [3.3; 19.7]	0.194
For Conquer	21.3 [6.5; 40.8]	22.1 [9.8; 45;8]	0.29

Bold = statistically significant differences.

**Table 3:** Central corneal thickness, and endothelial cell count and loss.

	eT2.2 HF	eT1.6 iaCOMICS HF	*p*-Values
**Central Corneal Thickness (CCT)**			
preop	538 ± 30	540 ± 32	0.806
day 1 postop	561 ± 42	563 ± 45	0.835
1 week postop	539 ± 33	542 ± 34	0.714
6 months postop	536 ±31	541 ± 33	0.55
**Endothelial Cell Count (ECC)**			
preop	2497 ± 257	2522 ± 283	0.659
6 months postop	2425 ± 292	2436 ± 331	0.867
**Endothelial Cell Loss (ECL)**	2.80 ± 7.28%	3.41 ± 8.25%	0.725

**Table 4:** Statistical significance of differences in Surgery Time, Effective Phaco Time, and Fluid Consumption CMP^®^ 2.8 mm low-fluidics phaco and eT2.2 enhanced fluidics phaco compared to high-fluidics eT2.2 HF and eT1.6 iaCOMICS HF phaco.

CMP^®^ (Cool Microincision Phaco) 2.8 mm with low fluidics			
*Phacopower (% max)*	*70*	Differences compared to eT2.2 HF *p*-values	Differences compared to eT1.6 iaCOMICS HF *p*-values
*Flow (mL/min)*	*20*		
*Vacuum (mmHG)*	*400*		
*Bottle height (cm)*	*70*		
**LOCS III**, Nuclear hardness	3 [2; 4]	**0.004**	**0.001**
**Surgery time**			
for Divide and Conquer	2 min 29 s [1 min 33 s; 4 min 14 s]	**<0.001**	**<0.001**
for Divide	47 s [28 s; 1 min 44 s]	**<0.001**	**0.004**
for Conquer	1 min 39 s [51 s; 3 min 13 s]	**<0.001**	**<0.001**
**Effective Phaco Time**			
for Divide and Conquer	9.4 [2.2; 32.3]	0.253	0.256
for Divide	3.6 [0.9; 11]	0.146	0.557
for Conquer	5.9 [0.2; 23]	0.071	0.117
**Fluid Consumption**			
for Divide and Conquer	40.9 [19.6; 80.1]	**<0.001**	**<0.001**
for Divide	8.2 [0; 26.2]	**0.023**	0.455
for Conquer	31.1 [13.1; 60.5]	**<0.001**	**<0.001**
**easyTip^®^2.2 with enhanced fluidics (eT2.2 EF)**			
*Phacopower (% max)*	*70*	Differences compared to eT2.2 HF *p*-values	Differences compared to eT1.6 iaCOMICS *p*-values
*Flow (mL/min)*	*35*		
*Vacuum (mmHG)*	*500*		
*Bottle height (cm)*	*70*		
**LOCS III**, Nuclear hardness	3 [1; 4.5]	**0.** **002**	0.001
**Surgery time (ST)**			
for Divide and Conquer	2 min 30 s [1 min 38 s; 4 min 26 s]	**<0.001**	**<0.001**
for Divide	49 s [23 s; 1 min 31 s]	**<0.001**	**0.002**
for Conquer	1 min 41 s [47 s; 2 min 59 s]	**<0.001**	**<0.001**
**Effective Phaco Time (EPT)**			
for Divide and Conquer	6.5 [1.6; 24]	**0.02**	**0.034**
for Divide	3.2 [1.4; 8]	**0.002**	0.053
for Conquer	3.0 [0.10; 16]	0.06	0.071
**Fluid Consumption (FC)**			
for Divide and Conquer	45.8 [21.3; 106.3]	**<0.001**	**<0.001**
for Divide	6.5 [3.3; 29.4]	**0.** **019**	**0.25**
for Conquer	36 [14.7; 94.8]	**<0.001**	**<0.001**

Bold = statistically significant differences.

## Data Availability

Data are contained within article.
